# Versatile Molding Process for Tough Cellulose Hydrogel Materials

**DOI:** 10.1038/srep16266

**Published:** 2015-11-05

**Authors:** Mutsumi Kimura, Yoshie Shinohara, Junko Takizawa, Sixiao Ren, Kento Sagisaka, Yudeng Lin, Yoshiyuki Hattori, Juan P. Hinestroza

**Affiliations:** 1 Division of Chemistry and Materials, Faculty of Textile Science and Technology, Shinshu University, Ueda 386-8567, Japan & Global Aqua Innovation Center, Shinshu University, Nagano 380-8553, Japan; 2 Department of Emerging Technology Research, Taiwan Textile Research Institute, 25162, Taiwan; 3 Department of Fiber Science and Apparel Design, Cornell University, 242 MVR Hall, Ithaca, NY, 14850.

## Abstract

Shape-persistent and tough cellulose hydrogels were fabricated by a stepwise solvent exchange from a homogeneous ionic liquid solution of cellulose exposure to methanol vapor. The cellulose hydrogels maintain their shapes under changing temperature, pH, and solvents. The micrometer-scale patterns on the mold were precisely transferred onto the surface of cellulose hydrogels. We also succeeded in the spinning of cellulose hydrogel fibers through a dry jet-wet spinning process. The mechanical property of regenerated cellulose fibers improved by the drawing of cellulose hydrogel fibers during the spinning process. This approach for the fabrication of tough cellulose hydrogels is a major advance in the fabrication of cellulose-based structures with defined shapes.

Common polymeric materials, which are derived from petroleum and natural gases, have been used in various fields around us. However, production of these plastics requires more fossil fuels and increases greenhouse gas emission. Recently, bio-based polymers derived from renewable biomass sources have been attracted a special attention as an alternative raw material of plastics. Cellulose, an organic renewable resource produced by plant synthesis from water and carbon dioxide, is the most abundant polymer on earth^1^. Cellulose is a linear and extended polysaccharide possessing a rod-like conformation, and its hydroxyl groups form strong hydrogen bonding networks with oxygen atoms on the same or neighboring cellulose chain. The stiff cellulose molecules can form highly crystalline domains through the stabilization by inter- or intramolecular hydrogen bonds. While the crystallization of cellulose exhibits good mechanical and thermal properties, the use of cellulose is limited due to its low solubility in most solvents. Non-derivatizing solvent systems for cellulose have been developed for producing regenerated cellulose fibers, films, and non-woven fabrics^2^. Since the report on the dissolution of cellulose in ionic liquids (ILs) by Swatloski *et al.*^3^, various ILs have been developed as non-derivatizing solvents for cellulose^4^,^5^,^6^,^7^,^8^. Ionic liquids enable dissolution of cellulose by disrupting and breaking hydrogen-bonding networks. Ohno and Fukaya *et al.* succeeded the preparation of a 10 wt% homogeneous cellulose solution under mild conditions by using *N*-ethyl-*N'*-methylimidazolium methylphosphonate ([C2mim][(MeO)(H)PO_2_])^9^,^10^.

Hydrogels have gained special attention as human and environmental friendly soft materials. Various natural and synthetic polymers are used as hydrophilic backbones of the three-dimensional network in hydrogels. Among these hydrophilic polymers, hydrogels made from biodegradable polysaccharides have been widely used for biomedical, food, and cosmetic applications^11^,^12^,^13^. Polysaccharide-based hydrogels can be formed through physical aggregation of polymer chains caused by hydrogen bonds, crystallization, helix formation, and complexation. The partial aggregation of polysaccharide chains acts as a network junction point in three-dimensional networks. Although cellulose gels are prepared through chemical crosslinking of cellulose derivatives or self-assembling of cellulose nanofibers, hydrogels prepared from homogeneous cellulose solutions are rare^14^,^15^,^16^,^17^,^18^,^19^,^20^,^21^. Kuga prepared cellulose gels by extracting calcium thiocyanate from a cellulose solution with methanol^22^. Regenerated cellulose hydrogels were also prepared from cellulose IL solution in 1-allyl-3-methylimidazolium chloride^23^,^24^. However, these cellulose hydrogels possess heterogeneous segregated structures in gels^11^,^12^,^13^,^14^,^15^,^16^,^17^,^18^,^19^,^20^,^21^,^22^,^23^,^24^. In this study, we report on a simple regeneration process of cellulose to enable the fabrication of shape-persistent, tough, and biodegradable hydrogel objects having various shapes. The hydrogen networks among cellulose backbones can be re-constructed by exchanging ILs from the homogeneous cellulose solutions. The regenerated cellulose materials contain unique hydrogen-bonded supramolecular structures^25^.

## Results and Discussion

**Figure 1:**
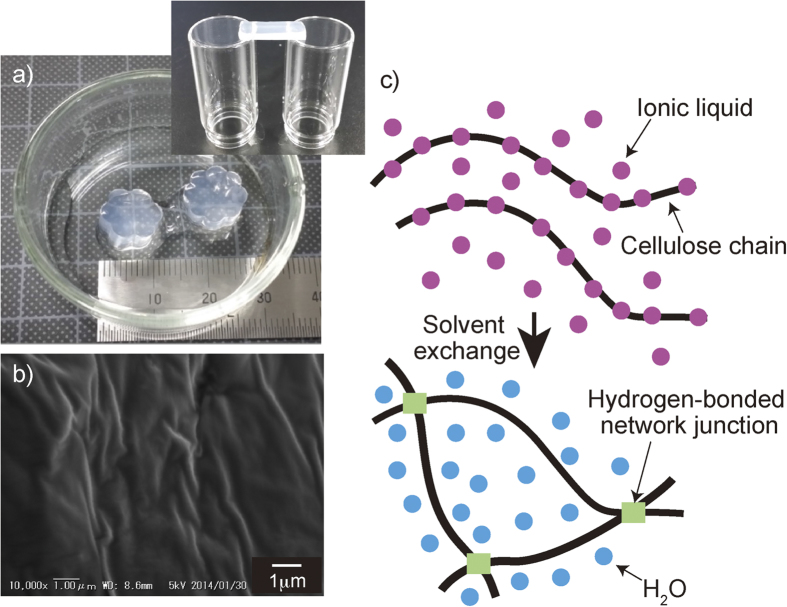
(**a**) Optical images of flower-shaped cellulose hydrogels prepared from 5 wt% IL solution of WP. The inset is an optical image of bridged cellulose hydrogel. (**b**) SEM image of the surface of dried cellulose hydrogel. (**c**) Schematic gelation process of the cellulose solution.

Two types of cellulose (microcrystalline cellulose (MCC) and wood pulp (WP)) with different degrees of polymerization (DPs) were dissolved within 5 hrs in [C2mim][(MeO)(H)PO_2_]. The color of cellulose solution did not change (Fig. S1), indicating that cellulose degraded a little during this dissolving process^9^. Cellulose was regenerated from the solution in a form of a gel by treating with methanol vapor. The structure is set uniformly by a spinodal decomposition-type phase separation during the treatment with methanol vapor, and the formed microcrystals of cellulose act as cross-linking points in the gels. After treatment with methanol vapor, the formed gels were washed with methanol for several times until no detection of ionic liquid in methanol analyzed by high performance liquid chromatography. Finally, methanol was exchanged with deionized water to obtain nearly transparent and self-standing cellulose hydrogels (Fig. 1a). The cellulose content in the resultant cellulose hydrogels was almost equal to the original concentration in cellulose solutions, and the original shape and volume did not alter during the solvent exchange process. The FT-IR spectra of dried cellulose gels did not show any peaks corresponding to ionic liquid, indicating the complete extraction of ILs with methanol and water from cellulose hydrogels (Fig. S2 and S7). Furthermore, the peak around 3300 cm^−1^ was broadened after the solvent exchange process, suggesting the change of hydrogen-network structure. Figure 1b shows a scanning electron microscopy (SEM) image of dried cellulose hydrogel. This image displays a smooth and homogeneous surface in which fibrous structures and small pores could not be observed. The X-ray diffraction patterns of dried cellulose gels showed only a broad hallow at 2θ = 20^o^, suggesting low crystallinity of cellulose in hydrogels (Fig. S3)^26^. The thermogravimetric analysis profile of the dried regenerated cellulose gels showed a low onset temperature for decomposition and high residual mass after the decomposition step relative to that of the parent cellulose (Fig. S4)^3^. Cellulose hydrogels containing 95–99% water can be fabricated using the solvent exchange of homogeneous cellulose solutions without the use of chemical crosslinking (Fig. 1c). Cellulose was completely dissolved in [C2mim][(MeO)(H)PO_2_] through the breaking of the hydrogen bonds among cellulose backbones, and the dissociated cellulose chains were partially aggregated during the solvent exchange process. Water was incorporated within the three-dimensional network of the hydrophilic cellulose chains containing hydrogen-bonded network junction points.

**Figure 2:**
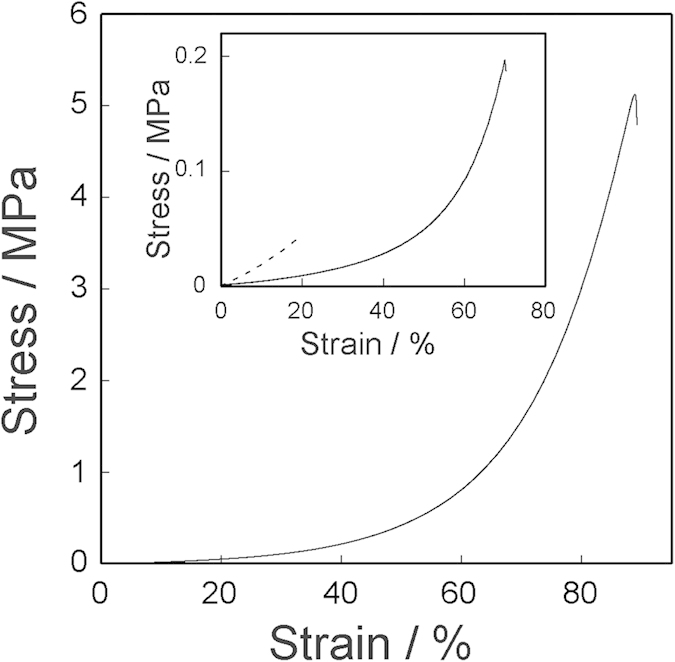
Stress-strain curve for 5 wt% WP cellulose hydrogel (water-content: 95 wt%) under uniaxial compression. The inset is the stress-strain curves of 1 wt% WP hydrogel (solid line; water-content: 99 wt%) and 5 wt% MCC hydrogel (dashed line; water-content: 95 wt%).

Figure 2 shows stress-stain curves of cellulose hydrogels under compression. Cellulose gels containing a 5 wt% WP break at a stress of 5.1 MPa, which is much higher than that of 5 wt% polysaccharide-based agarose hydrogels (0.03 MPa). The mechanical strength of cellulose hydrogels strongly depends on the concentration and DP of cellulose. Cellulose gels can sustain a strain of 60% upon compression without failure. Agarose hydrogel was melted in near-boiling water, while cellulose gels did not show liquid-gel transition. Moreover, the mechanical property of cellulose gel remained unaltered after treatment with boiled water, indicating the excellent thermal stability of cellulose hydrogels. The cellulose hydrogels exhibited a shape persistent when immersed in water-miscible organic solvents (tetrahydrofuran (THF), methanol, dimethylformamide (DMF), dimethylsulfoxide (DMSO), and acetone) and water-immiscible oils (silicone oil and soybean oil) (Fig. S5). The network of cellulose chains maintained its form after replacement with organic solvents. After replacement of water with THF, the cellulose gels were allowed to stand in air at 20 °C to evaporate THF. The gels were shrunk into dried solids while maintaining their original shapes. The resultant solids did not recover their original dimensions after soaking in water. Hydroxyl groups in cellulose chains came closer during gel shrinkage and form a dense hydrogen network in the solid. The formation of dense hydrogen network prevented swelling after soaking the dried solids in water.

**Figure 3:**
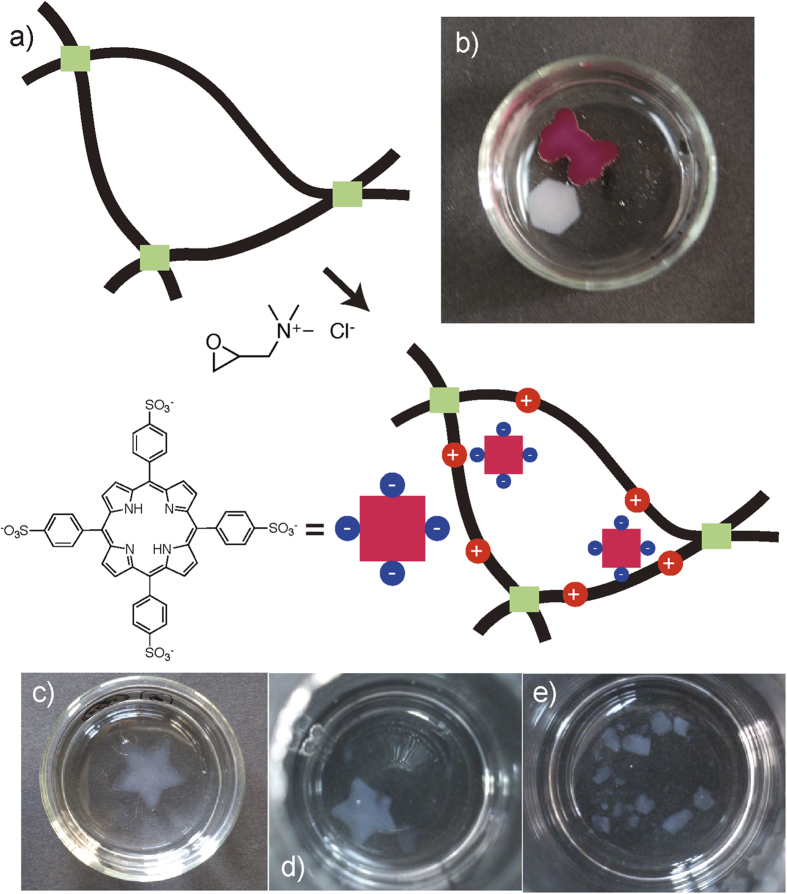
(**a**) Schematic modication process of the cellulose hydrogel with EPTAC and TPPS. (**b**) Picture of modified and non-modified cellulose gels stained with TPPS. Pictures of star-shaped cellulose hydrogel before (**c**) and after being immersing a solution of *cellulase* for (**d**) 30 min and (**e**) 2 hrs at 50 °C.

Cellulose hydrogels are reacted with 2,3-epoxypropyltrimethylammonium chloride (EPTAC) in 0.1 M NaOH aqueous solution at 60 °C for 4 hrs (Fig. 3a)^27^,^28^,^29^. The reaction of the hydroxyl groups of cellulose with ammonium epoxides introduces positive charges in three-dimensional network of cellulose chains without changing the size and shape of the hydrogel. The X-ray photoelectron spectroscopy measurement of dried cationized cellulose gel showed a weak peak at 398.9 eV, revealing the presence of nitrogen^30^. The whole cellulose gel possessing positive charges was rigidly stained by immersing it into aqueous solution of tetrakis(4-sulfonatophenyl)porphyrin (TPPS) (Fig. 3b). Negative charged TPPS was incorporated within the three-dimensional network of the modified cellulose chains through electrostatic interaction. Figure 3c–e show the decomposition of the cellulose gel by *cellulase* (2.1 mg/ml) in a citrate buffer at 50 °C. *Cellulase* can break down cellulose chains into monosaccharide or shorter polysaccharides through the hydrolysis of 1,4-β-D-glycosidic linkages^31^. The cellulose gels were collapsed into small pieces by the enzymatic activity of *cellulose* and the mechanical strength of cellulose hydrogels significantly decreased by the treatment with *Cellulase* (Fig. S6), implying the biodegradability of cellulose hydrogels.

**Figure 4:**
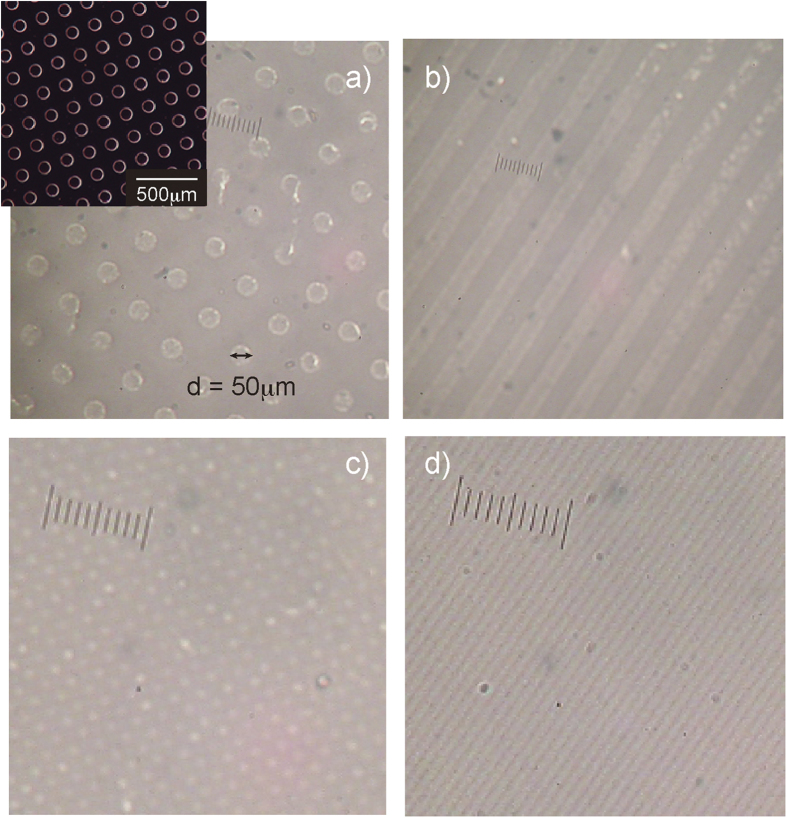
Optical images of patterned cellulose hydrogels; (**a**) holes (the inset is the pattern of mold), (**b**) lines (line width: 50 μm), (**c**) holes (diameter: 5 μm) and (d) lines (line width: 5 μm).

Cellulose hydrogels composed of rod-like and stiff cellulose chains maintain their original volumes and shapes with keeping good mechanical properties under various conditions, whereas conventional hydrogels exhibit drastic volume changes in response to several external stimuli such as temperature, solvent changes, pH, and light. We fabricated patterned cellulose gels by the gelation process of a cellulose solution onto silicon molds. Imprint lithography has been demonstrated as a high-resolution and cost-effective patterning technique for the fabrication of devices for electrical, optical, and biomedical applications. The patterns of molds or stamps are transferred to targets through thermal embossing or UV curing processes. Several research groups have examined patterning techniques toward the creation of micrometer-sized three-dimensional architectures in hydrogels^32^,^33^,^34^. A homogeneous cellulose solution was placed onto the patterned mold surface and degassed under vacuum to fill patterns in the mold with cellulose solution. After treating with methanol vapor and washing with methanol/water, the cellulose hydrogels were peeled from the surface of the silicon mold. Figure 4 shows the optical images of micrometer-sized structures, such as lines and circular cavities, on cellulose hydrogels. The imprinted patterns on the cellulose hydrogel were observed to be identical in size to those on the silicon mold.

**Figure 5:**
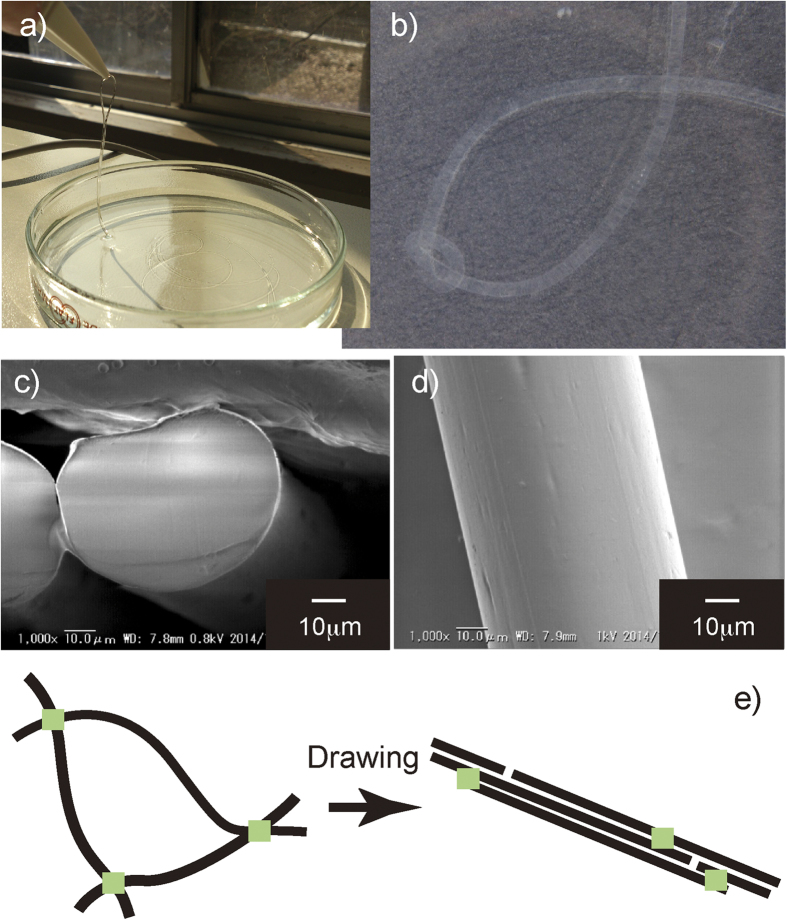
(**a,b**) Cellulose hydrogel fiber and knotted fiber in water. (**c**) Cross-sectional and (**d**) side SEM images of dried cellulose fibers. (**e**) Schematic illustration of cellulose orientation during drawing process.

When the heated cellulose IL solution is extruded from the spinneret, the fibrous cellulose gel is formed through cooling and exposure to methanol vapor within air gap between the spinneret and the coagulation bath. The cellulose IL solution spun continuously until the end of solution supply and transparent cellulose hydrogel fiber was obtained after washing with water (Fig. 5a). Regenerated cellulose fibers have been commercially produced from cellulose xanthate or cuprammonium solutions of cellulose^35^. These processes include toxic chemical treatments to prepare a spinnable solution, which cause serious pollution. To reduce the environmental impact of the production process of regenerated cellulose fibers, non-derivatizing solvent systems including ILs for cellulose have been developed^36^,^37^,^38^,^39^,^40^. However, the reported regenerated cellulose fibers spun from cellulose IL solutions exhibited interior mechanical properties relative to the regenerated cellulose fiber (Lyocell) from *N*-methyl-morpholine-*N*-oxide/water system^41^. The cellulose IL solution was spun by a dry jet-wet spinning process using a methanol coagulation bath, and the mechanical properties of regenerated cellulose fibers were analyzed. In the previous studies, the cellulose IL solutions were coagulated within water to form regenerated cellulose fibers^36^,^37^,^38^,^39^,^40^. Whereas the resultant as-spun fibers contained a highly content of water (93%), the fiber formed a knot suggesting a sufficiently high mechanical property of cellulose hydrogel fiber in water (Fig. 5b). The dried cellulose fiber exhibited a uniform circular diameter with a smooth and homogeneous surface and did not contain internal pores (Fig. 5c,d). The circular section of cellulose fibers was different from that of conventional viscose fibers, which have a lobulated skin-core structure^42^,^43^. This confirmed homogenous coagulation without the formation of a skin layer. Above 95% of IL in the dope solution was recovered from the coagulation bath solution by evaporation, and the recovered IL was recycled to be reused for cellulose dissolution.

The tensile strength and breaking elongation of the as-spun cellulose fibers were 0.5 cN/dtex and 15.2%, respectively. The as-spun cellulose fiber exhibited a low tensile strength relative to the commercial viscose rayon^21^, presumably due to the low crystallization degree of cellulose. When the cellulose gel fiber was drawn within the methanol coagulation bath by increasing the take-up speed of the godet (draw ratio (D_R_) = 10.5), the drawn fibers showed a tensile strength of 4.0 cN/dtex and a modulus of 28.1 GPa with a low elongation of 3.9%, which are almost similar to those of Lyocell^41^,^43^. The enhancement of mechanical properties for the drawn fiber implies the orientation of cellulose chains along the filament axis by the drawing of cellulose gel fibers (Fig. 5e). The cellulose fibers were heated at 1000 °C for 2 hr under an argon atmosphere to obtain the carbon fibers. Although the carbonization of the as-spun cellulose fibers provided a powdered sample, the drawn fibers maintained the fibrous morphology with a char yield of 18% (Fig. S8). The Raman spectrum of carbonized cellulose fiber showed two peaks at 1323 and 1590 cm^−1^, which correspond to the disordered (D) and graphitic (G) bands, respectively (Fig. S9)^44^. The appearance of G band indicates the formation of graphenes by the carbonization of oriented cellulose assemblies in the drawn fibers. The cellulose hydrogel fibers were easy to handle and could be used to construct unique architectures possessing spatial arrangement of gel fibers through knitting and weaving. Furthermore, gel fibers can incorporate various water-soluble nanomaterials such as nanoparticles and proteins, within the three-dimensional network of cellulose chains. The incorporated nanomaterials can communicate with the outer environment through open gates of cellulose networks. The fabrics assembled with several functional cellulose gel fibers may be a useful platform for multifunctional water-based devices.

## Conclusion

We have demonstrated the molding process of tough cellulose materials by the stepwise solvent exchange from a homogeneous IL solution of cellulose. The cellulose hydrogels maintain their shapes under changing temperature, pH, and solvents. The micrometer-scale patterns on the mold were precisely transferred onto the surface of cellulose hydrogels. We also succeeded in the spinning of cellulose hydrogel fibers through a dry jet-wet-spinning process. Molding of renewable cellulose resources into various shapes is a promising technology to realize bio-based products. The tough cellulose hydrogels and fibers may open new application possibilities such as scaffolds for tissue engineering, micro-reactors using microfluidics and advanced membranes for water recycling/reuse.

## Method

Preparation of Cellulose Hydrogels: Cellulose powders were dissolved in [C2mim][(MeO)(H)PO_2_] (Kanto Chemical) at 60 °C with stirring under vacuum. Clear and viscose cellulose solutions were cast into various shaped molds and the molds were allowed to stand in sealed vessels containing methanol for 2 days at room temperature. After 2 days, the formed cellulose gels were immersed into water for 1 day.

Dry Jet-Wet Spinning: Cellulose fibers were fabricated using a custom-built wet-spinning apparatus to demonstrate the continuous fiber spinning process. The cellulose dope (7 wt% WP solution) was transferred to a spinning pack, and the temperatures of spinning pack were maintained at 60 °C during spinning. The filament is first extruded from a spinneret with a hole diameter of 0.2 mm (length-to-diameter ratio = 2.0) through an air gap (distance between the nozzle and methanol bath = 5 cm). The extruded polymer solution was solidified to form continuous fibers within a methanol coagulation bath. The resulted cellulose fibers were washed with methanol and water. The regenerated cellulose fibers were converted to carbon fiber by heating at 900 °C for 2 h under an inert gas atmosphere.

## Additional Information

**How to cite this article**: Kimura, M. *et al.* Versatile Molding Process for Tough Cellulose Hydrogel Materials. *Sci. Rep.* **5**, 16266; doi: 10.1038/srep16266 (2015).

## Supplementary Material

Supplementary Information
