# Supplementary material for: Versatile Molding Process for Tough Cellulose Hydrogel Materials
Source: Sci Rep. 2015 Nov 5;5:16266. doi: 10.1038/srep16266 (PMC4633679; doi:10.1038/srep16266)
Supplement: Supplementary Information [file srep16266-s1.pdf]

Supporting Information

Versatile Molding Process for Tough Cellulose Hydrogel Materials

Mutsumi Kimura, Yoshie Shinohara, Junko Takizawa, Sixiao Ren, Kento Sagisaka, Yudeng Lin, Yoshiyuki Hattori and Juan P. Hinestroza*

Experimental Part

Material: Ionic liquid [C2mim][(MeO)(H)PO_2] was purchased from Kanto Chemical and used as supplied. Microcrystalline cellulose (MCC, DP = 270) was purchase from Wako Chemicals, and wood pulp (WP, DP = 680) was provided from Taiwan Textile Research Institute. The viscosity-average degrees of polymerization (DP) of MCC and WP were measured using an Ubbelohde viscometer in cupriethylenediamine hydroxide solution.[1] Tetrakis(4-sulfonatophenyl)porphyrin (TPPS) and *Cellulase* from *Aspergillus niger* were purchased from Tokyo Kasei Chemicals. 2,3-Epoxypropyltrimethylammonium chloride was purchased from Aldrich. All reagents were analytic grade and were used without further purification. Silicon mold having several patterns (Trial Mold (2) 1-50 μm) was purchased from Kyodo International Inc.

Ref) W. Brown, R. A. Wiskston, *Eur. Polym. J.*, **1965**, *1*, 1.

Characterization: Surface morphology and average fiber diameter of cellulose gels and fibers were characterized using scanning electron microscopy (SEM) (Keyence VE-8800). Thermogravimetry analyses (Seiko Instrument TGA/DTA 6200) was used to characterize the thermal properties of cellulose gels. A piece of dried cellulose gel (2-5 mg) was placed in an aluminum sample pan and heated from 30 to 500°C at 10°C/min under a N_2 atmosphere. IR spectra were obtained on a Shimazu IR Prestige-21 with DuraSample IR II. HPLC analyses were carried out with a JASCO HPLC system (pump 1580, UV detector 1575, refractive index detector 930) and a silica gel column in methanol as an eluent at 25 ^{o}C (1.0 ml/min). The crystalline structure of the samples was analyzed using wide-angle X-ray diffraction (Rigaku XRD-DSC) with Cu Kα radiation. The chemical composition of cationized cellulose gels was analyzed using X-ray photoelectron spectroscopy (Kratos Analytical Axis Ultra). Stress-strain curves for cellulose gels under uniaxial compression and cellulose regenerated fibers were obtained on a Shimazu EZ-test instrument equipped with a 500 N load cell. Raman spectra were obtained on a HaloLab 5000 spectrophotometer at an excitation wavelength of 532 nm. Water contents of cellulose hydrogels and as-spun fibers were determined by weight difference between before and after drying of samples.

Preparation of Agarose gels: Reference agarose gels were prepared by dissolving the agarose powder (Wako Chemicals). The 0.5g agarose powder was dispersed in 10ml deionized water and the dispersed solution was heated to near boiling point. The melted agarose was allowed to cool for the formation of hydrogels.

Modification of Cellulose gels with EPTAC: Cellulose gels were immersed into 0.1 M NaOH aqueous solution (10 ml). 0.1g 2,3-Epoxypropyltrimethylammonium chloride was added to the solution and heated at 60 ^{o}C for 4 hrs. After 4 hrs, the cellulose hydrogels were washed with water for several times and stored in deionized

water. The modified cellulose gels were immersed into 10μM aqueous solution of tetrakis(4-sulfonatophenyl)porphyrin (TPPS) for 10 min, and washed with water to remove physically absorbed TPPS within gels. When non-modified cellulose gels were immersed TPPS solution, the gel did not stain with TPPS.

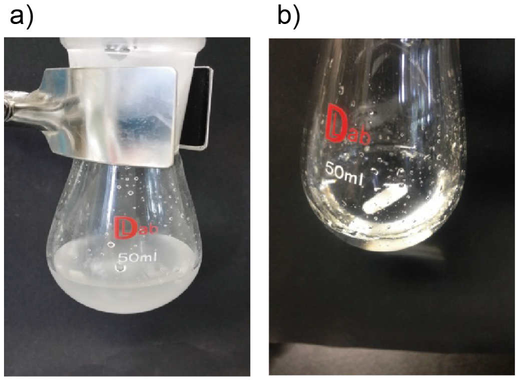

Fig. S1 Pictures of 5 wt% cellulose solution a) before and b) after heat treatment for 5 hrs at 60°C with stirring under vacuum in in [C2mim][(MeO)(H)PO_2].

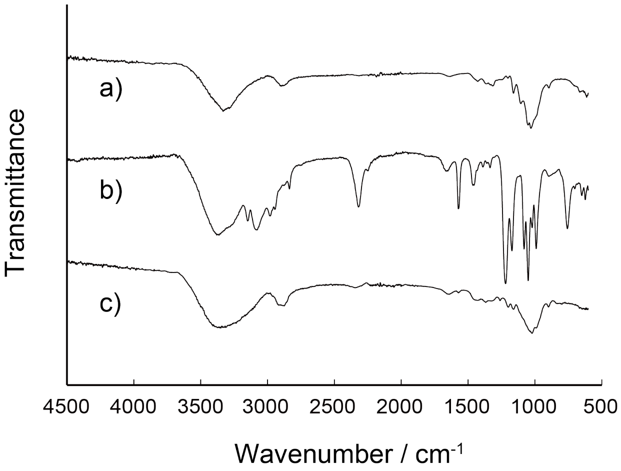

Fig. S2 FT-IR spectra of a) original WP, b) IL solution of WP, and c) dried cellulose gel.

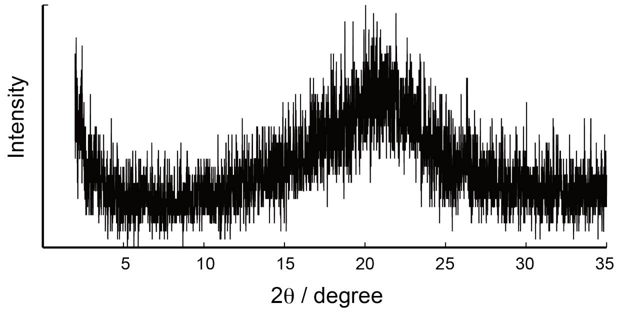

Fig. S3 Powder X-ray diffraction pattern of dried cellulose hydrogel.

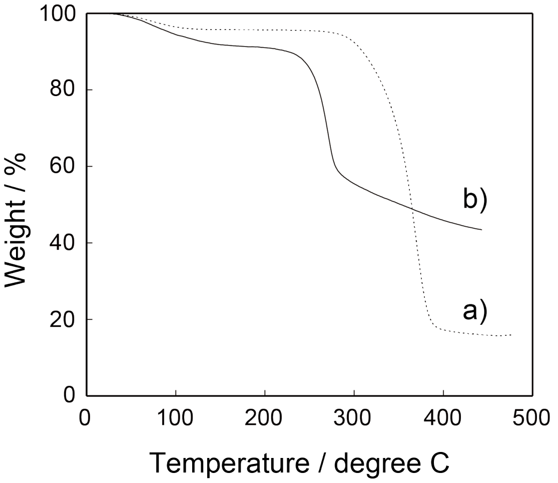

Fig. S4 TGA profiles of a) original WP and b) dried cellulose gel. Samples were heated at 10 ^{o}C under a nitrogen atmosphere.

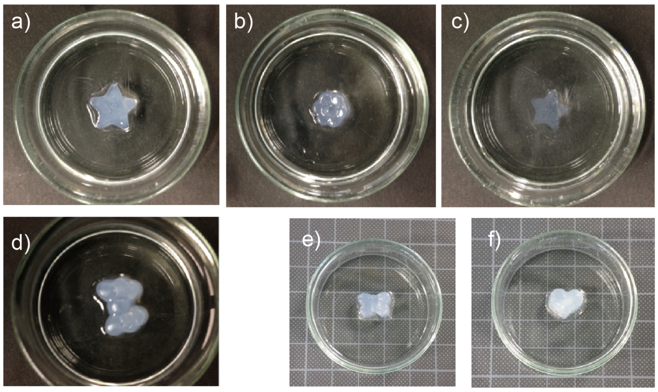

Fig. S5 Pictures of 1 wt% WP hydrogels after immersing in a) THF, b) DMF, c) DMSO, d) acetone, e) silicone oil and f) soybean oil at room temperature.

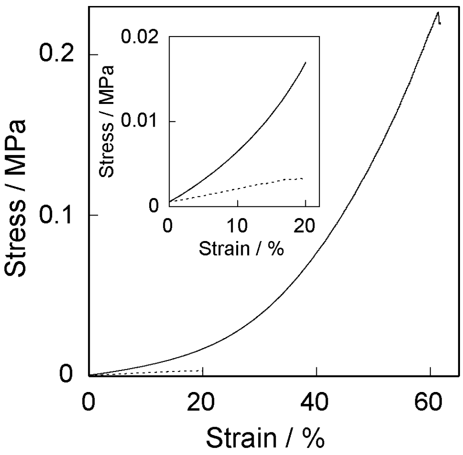

Fig. S6 Stress-strain curve for 1 wt% WP cellulose hydrogel (water-content: 99wt%) under uniaxial compression before (solid line) and after being immersing a solution of *cellulase* for 3hrs at 50°C (dotted line).

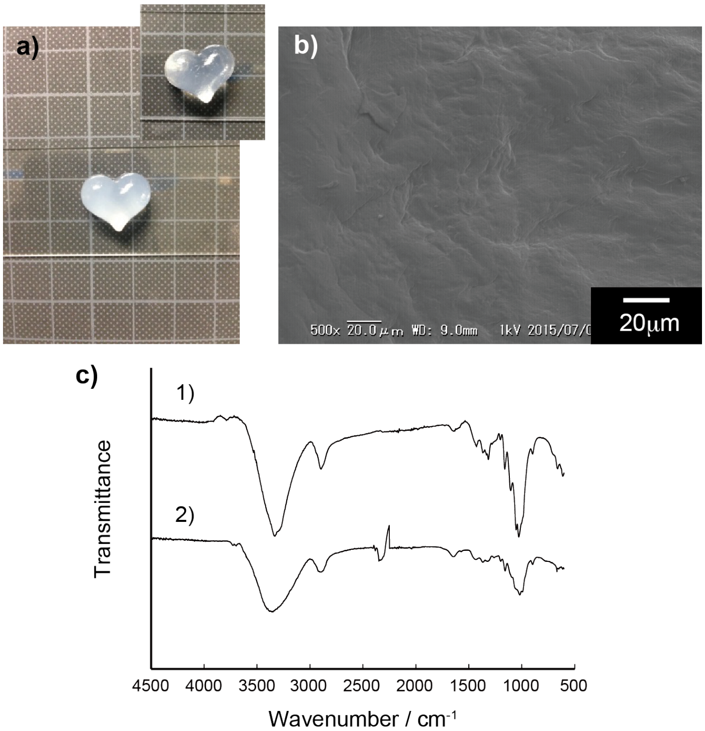

Fig. S7 Optical images of heart-shaped cellulose hydrogels prepared from 1 wt% IL solution of MCC. The inset is a picture of 1 wt% MCC hydrogels after immersing in DMF. b) SEM image of the surface of dried cellulose hydrogel. c) FT-IR spectra of 1) original MCC and 2) dried cellulose gel.

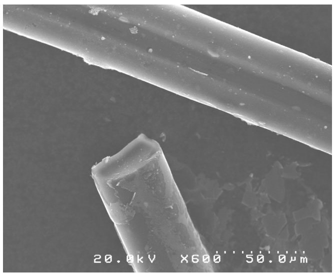

Fig. S8 FE-SEM image of carbon fibers for the drawn cellulose fibers after being carbonized at 1000 ^{o}C.

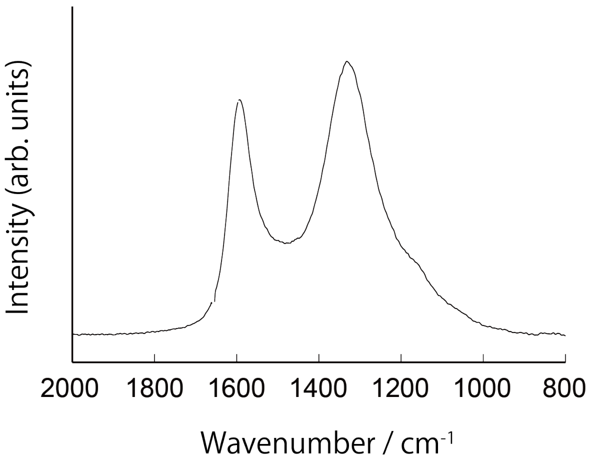

Fig. S9 Raman spectrum of carbon fiber.
